# Control of Oxidative Stress and Generation of Induced Pluripotent Stem Cell-like Cells by Jun Dimerization Protein 2

**DOI:** 10.3390/cancers5030959

**Published:** 2013-07-26

**Authors:** Shyh-Shin Chiou, Sophie Sheng-Wen Wang, Deng-Chyang Wu, Ying-Chu Lin, Li-Pin Kao, Kung-Kai Kuo, Chun-Chieh Wu, Chee-Yin Chai, Cheng-Lung Steve Lin, Cheng-Yi Lee, Yu-Mei Liao, Kenly Wuputra, Ya-Han Yang, Shin-Wei Wang, Chia-Chen Ku, Yukio Nakamura, Shigeo Saito, Hitomi Hasegawa, Naoto Yamaguchi, Hiroyuki Miyoshi, Chang-Sheng Lin, Richard Eckner, Kazunari K. Yokoyama

**Affiliations:** 1 Division of Hematology-Oncology, Department of Pediatrics, Kaohsiung Medical University Hospital, Kaohsiung 807, Taiwan; E-Mails: patapata007@gmail.com (C.-Y.L.); p920271@gmail.com (Y.-M.L.); 2 Department of Pediatrics, Faculty of Medicine, School of Medicine, Kaohsiung Medical University, 807 Kaohsiung 807, Taiwan; 3 Department of Gastroenterology, Kaohsiung Medical University Hospital, Kaohsiung 807, Taiwan; E-Mails: swang910@gmail.com (S.S.-W.W.); dechwu@yahoo.com (D.-C.W.); silviaw@hotmail.com.tw (S.-W.W.); 4 School of Dentistry, College of Dentistry, Kaohsiung Medical University, Kaohsiung 807, Taiwan; E-Mail: chulin@cc.kmu.edu.tw; 5 Graduate Institute of Medicine, College of Medicine, Kaohsiung Medical University, 807 Kaohsiung 807, Taiwan; E-Mails: kaolp@yahoo.com.tw (L.-P.K.); stevelin@kmu.edu.tw (C.-L.S.L.); kenlywu@hotmail.com (K.W.); R991046@cc.kmu.edu.tw (C.-C.K.); saict1@maple.ocn.ne.jp (S.S.); changshen.lin@gmail.com (C.-S.L.); 6 Department of Surgery, Cancer Center, Kaohsiung Medical University Hospital, Kaohsiung 807, Taiwan; E-Mails: kuoksfo@yahoo.com.tw (K.-K.K.); sal9522059@yahoo.com.tw (Y.-H.Y.); 7 Department of Pathology, Kaohsiung Medical University Hospital, Kaohsiung 807, Taiwan; E-Mails: lazzzwu@gmail.com (C.-C.W.); cychai@kmu.edu.tw (C.-Y.C.); 8 RIKEN BioResource Center, Tsukuba, Ibaraki 305-0074, Japan; E-Mails: yukionak@brc.riken.jp (Y.N.); miyoshi@brc.riken.jp (H.M.); 9 Saito Laboratory of Cell Technology, Yaita, Tochigi 329-1571, Japan; 10 Graduate School of Pharmaceutical Science, Chiba University, Chiba 260-8675, Japan; E-Mails: jin1103@graduate.chiba-u.jp (H.H.); nyama@faculty.chiba-u.ac.jp (N.Y.); 11 Department of Biochemistry & Molecular Biology, UMDNJ-New Jersey Medical School, Newark, NJ 07101, USA; E-Mail: rechard_eckner@yahoo.com

**Keywords:** anti oxidation, iPSC-like cells, Jun dimerization protein 2, medulloblastoma, ROS, oxidative stress

## Abstract

We report here that the Jun dimerization protein 2 (JDP2) plays a critical role as a cofactor for the transcription factors nuclear factor-erythroid 2-related factor 2 (Nrf2) and MafK in the regulation of the antioxidants and production of reactive oxygen species (ROS). JDP2 associates with Nrf2 and MafK (Nrf2-MafK) to increase the transcription of antioxidant response element-dependent genes. Oxidative-stress-inducing reagent led to an increase in the intracellular accumulation of ROS and cell proliferation in *Jdp2* knock-out mouse embryonic fibroblasts. In *Jdp2*-Cre mice mated with reporter mice, the expression of JDP2 was restricted to granule cells in the brain cerebellum. The induced pluripotent stem cells (iPSC)-like cells were generated from DAOY medulloblastoma cell by introduction of JDP2, and the defined factor OCT4. iPSC-like cells expressed stem cell-like characteristics including alkaline phosphatase activity and some stem cell markers. However, such iPSC-like cells also proliferated rapidly, became neoplastic, and potentiated cell malignancy at a later stage in SCID mice. This study suggests that medulloblastoma cells can be reprogrammed successfully by JDP2 and OCT4 to become iPSC-like cells. These cells will be helpful for studying the generation of cancer stem cells and ROS homeostasis.

## 1. Introduction

Oxidative stress can be induced by a vast range of agents, including xenobiotic, drugs, heavy metals, and ionizing radiation, and can lead to the generation of reactive oxygen species (ROS) and electrophiles. ROS often contribute to diseases such as cancer, cardiovascular complications, acute and chronic inflammation, and neurodegenerative condition [1,2]. Therefore, cellular defense mechanisms are required to control constantly the levels of ROS and to prevent their accumulation. Many antioxidant and/or detoxification enzymes, such as NADPH: quinone oxidoreductase 1 (NQO1) [3,4], glutathione *S*-transferase (GST) [5], and heme oxygenase-1 (HO-1) [6] contribute to cellular defenses systems against ROS. The coordinated induction of the genes encoding the Phase II detoxification and antioxidant enzymes is governed by the core sequence 5'-G/A TGACNNNGC-3' located in their gene regulatory regions, and is termed the antioxidant responsive element (ARE) [7]. Nuclear factor-erythroid 2-related factor 2 (Nrf2) is the key factor responsible for binding to AREs. This basic leucine zipper (bZIP) transcription factor belongs to the cap “n” collar (CNC) protein family (encompassing Nrf1, Nrf2, Nrf3, and p45 NF-E2) and forms heterodimer complexes with a small Maf protein (either MafG, MafK, or MafF), which provide high-affinity binding to AREs [5,8]. The Nrf2-ARE axis is absolutely essential for the induction of genes encoding antioxidant and detoxification enzymes, and contributes to the regulation of cell growth, differentiation, and survival after oxidative stress.

The c-Jun dimerization protein 2 (JDP2) is a bona fide member of the AP-1 transcription factor family [9,10] and is a bZIP repressor protein that is highly expressed in the brain and lung [11]. JDP2 can homodimerize or form heterodimers with other AP-1 family members [9,10,11,12]. The mechanism by which JDP2 represses AP-1 transcription involves competition for DNA binding, inactive heterodimer formation [9], indirect recruitment of histone deacetylase 3 [13], nucleosome assembly activity, inhibition of histone acetylation [14], and potential competition with JNK phosphorylation [15]. The knockout (KO) of *Jdp2* gene affects adipocyte differentiation [16], resistance to replicative senescence [17], cell cycle arrest and regulation of cyclin A2 [18]. Retroviral activation of alternative *Jdp2* in T cell lymphomas of mice has been reported, providing the strong evidence for a gain-of-function of Jdp2 in cancer development in the hematopoietic system [18]. Recent studies of tumor cells have demonstrated that JDP2 is a tumor suppressor [19,20], suggesting that genomic alterations might be the underlying cause of cancer development. However, some studies have shown that JDP2 can potentiate cancer cell growth [21,22]. It is not known whether these amplifications of JDP2 produce abundant amounts of normal JDP2 protein or the truncated JDP2 mRNAs, which are thought to be an oncogene [18]. Other bZIP factors, such as JunD, PMF-1, and ATF4, bind to the ARE and can regulate ARE-driven transcription [23,24]. The small Maf proteins can dimerize with CNC factors, such as Nrf2, and with other bZIP factors, including Fos, FosB, Bach 1, and Bach 2, via their leucine zipper domain [25]. Because JDP2 is also a member of the bZIP family of transcription factors, we examined whether JDP2 binds to Maf-family and/or Nrf2 proteins, and whether it can regulate ARE-dependent genes encoding antioxidant and detoxification enzymes.

Somatic cells have been reprogrammed successfully into induced pluripotent stem cells (iPSCs) by ectopic overexpression of the transcription factors OCT4, SOX2, KLF4, and c-MYC [26]. Other sets of transcription factors have also been reported to induce iPSCs from somatic cells [27,28]. Similar approaches have been used for the reprogramming of cancer cells into induced pluripotent cancer cells (iPCCs) by different sets of transcription factors [29,30,31,32]. Both types of pluripotent cells, iPSCs and iPCCs, share characteristic features with each other as well as with embryonic stem cells (ESCs) [33]. During reprogramming of somatic or cancer cells, ROS are generated by metabolic stress, and increased ROS levels lead to DNA damage, cell senescence, and apoptosis. ROS may hinder the survival of reprogrammed cells, as suggested by observations of increased iPSCs generation during hypoxia [34,35]. In addition, oxidative stresses repress the ability to generate or maintain iPSCs and human ESCs (hESCs) [36], suggesting that ROS generation by reprogramming factors is unfavorable for generating iPSCs.

Here we report that JDP2 indeed associates with the ARE and acts as a newly identified key cofactor of the Nrf2-MafK complex to regulate ARE-mediated gene expression and ROS production. In *Jdp2*-Cre mice, Jdp2 was specifically expressed in granule cells in the cerebellum. We reprogrammed medulloblastoma cells [37] to generate iPSC-like cells using the transduction of lentivirus-encoded JDP2 and OCT4 to characterize the reprogrammed medulloblastoma iPSC-like cells. We also discuss the role of JDP2 in cancer cell reprogramming and ROS homeostasis. Our results provide evidence that JDP2 plays a critical role in the cellular adaptive response to ROS and electrophiles generated by various cellular stimuli and in the generation of cancer progenitor cells with the phenotypes of stem cells and cancer cells.

## 2. Experimental Section

### 2.1. Reagents, Cell Culture, and Plasmid Preparation

Antibodies against Nrf2 (C-20), NF-E2p18 (MafK; C-16), α-tubulin (B-7), β-actin (AC-15), HO-1 (H-105), and NQO1 (C-19) were from Santa Cruz Biotechnology Inc. (Santa Cruz, CA, USA). The JDP2 hetero antibodies were from Abcam (ab40916, lot #469784; Cambridge, UK), and kindly gifted by Dr. Aronheim [9], and the monoclonal antibodies against JDP2 were described elsewhere [10,11]. Acetone, tricarbonyldichlororuthenium(II) dimer (CORM), protease inhibitor mixture, hydrogen peroxide (H_2_O_2_, 30%) and all the other chemicals were obtained from Sigma-Aldrich (St. Louis, MO, USA). Sulforaphane (SFN) was purchased from the LKT Laboratory (St. Paul, MN, USA). The human DAOY medulloblastoma cell line, HeLa CD4+ and 293T cells were obtained from ATCC (Manassas, VA, USA) and were cultured in Dulbecco’s Modified Eagle Minimal Essential medium (DMEM)-Ham’s F-12 (Invitrogen, Grand Island, NY, USA). Wild-type (WT) and *Jdp2* KO mouse embryonic cells (MEFs) were prepared as described elsewhere [11]. A plasmid of mouse *Jdp2* and its GST-fusion deletion mutants were constructed as described previously [13,14]. The full-length plasmids pcDNA3–rat Nrf2 and pcDNA3-rat MafK were kindly provided by Dr. T Nguyen (Schering-Plough Research Institute, Kenilworth, NJ, USA). All recombinants were confirmed by DNA sequencing.

### 2.2. Measurement of H_2_O_2_ Concentrations in Culture Medium

Hydrogen peroxide concentrations in the culture medium were measured by ferrous oxidation of xylenol orange (FOX) assay [38]. Samples of culture media were added at specific intervals to FOX reagent, which comprised 100 mM xylenol orange, 250 mM ammonium ferrous sulfate, 100 mM sorbitol, and 25 mM H_2_SO_4_. Changes in absorbance at 560 nm were measured.

### 2.3. Preparation of Hydrogen Peroxide

Hydrogen peroxide (30% v/v) was diluted to a concentration of 100 mM in distilled water. The precise concentration of hydrogen peroxide was determined using the titanium oxide method [39], in which the molar coefficient of a titanium oxide-hydrogen peroxide complex is assumed to be 750 M^−1^ cm^−1^ at 405 nm. Briefly, 160 μL of hydrogen peroxide solution (prepared as described above) were added to a mixture of 30 mL of titanium sulfate and 50 mL of 20% (v/v) hydrogen sulfate. The resulting mixture was stirred at room temperature for 15 min, and the precise concentration of hydrogen peroxide was calculated from the absorbance at 405 nm.

### 2.4. Analysis of 7,8-Dihydro-8-oxo-8-2′-deoxyguanosine (8-oxo-dGuo), Glutathione, and Cellular ROS

8-Oxo-dGuo and glutathione concentrations were measured using liquid chromatography-mass spectrometry, as described elsewhere [40]. To measure the net intracellular accumulation of ROS, a fluorescent probe species (2',7'-dichlorofluorescein, DCF-DA; Molecular Probes, Eugene, OR, USA) was used. After 2 h of treatment with H_2_O_2_ or SFN, cells were washed twice with HBSS solution (Gibco, Carlsbad, CA, USA) and loaded with 10 mmol/L of DCF-DA in a 5% CO_2_ incubator kept at 37 °C. After 30 min, the cells were washed twice with HBSS, suspended in complete medium, and examined under a microscope. The number of DCF-stained cells was calculated in an area of 8.75 mm^2^.

### 2.5. Transient Transfection and Luciferase Reporter Assay

WT and *Jdp2* KO MEFs (1 × 10^5^ cells) were plated into each well of 12-well plates and cultured for 24 h. The cells were then cotransfected with the indicated amount of pGL4-hQR25-firefly luciferase reporter and pGL4-TK plasmid encoding *Renilla* luciferase (Promega, Madison, WI, USA) using the Effectene transfection reagent kit (Qiagen, Valencia, CA, USA) or Lipofectamine 2000 (Invitrogen Co., Grand Island, NY, USA). To induce overexpression, cells were cotransfected with pcDNA3 encoding Nrf2, MafK, or JDP2, respectively. The total amount of transfected DNA was kept constant at 1 μg/well by the addition of pcDNA3. After 24 h of incubation, the cells were incubated in the presence or absence of 10^–6^ M TPA or 5 × 10^−6^ M SFN in DMSO (or DMSO alone, as a control) for 24 h. The activities of luciferase and *Renilla* were measured in a illuminometer (Berthold Technologies Gmbh and Co. KG, Bad Wildbad, Germany) using the Dual-Luciferase Reporter Assay System (Promega). Luciferase activity values were normalized to transfection efficiency.

### 2.6. Western Blot Analysis

Cells were harvested using a modified RIPA buffer (10 mM Tris-HCl, pH 8.0, 150 mM NaCl, 1 mM EDTA, 0.1% NP-40, 1% deoxycholate, 50 mM sodium fluoride, 50 mM sodium orthovanadate, and 1 mM PMSF) and a protease inhibitor cocktail (Nacalai Tesque, Kyoto, Japan). The preparation of cell lysates, SDS-PAGE (8% or 10% gel) and western blotting were performed as described elsewhere [11,13].

### 2.7. Chromatin Immunoprecipitation (ChIP) Assay

ChIP assays were performed as described by Kotake *et al.* [41] with modification of the washing conditions. The immunoprecipitated protein-DNA complexes were washed twice with binding buffer (10 mM HEPES, pH 7.9, 10 mM Tris-HCl, pH 7.9, 12.5% glycerol, 0.25% NP-40, 0.5% Triton X-100, 0.24 M NaCl, 0.75 mM MgCl2, 1.1 mM EDTA, and protease inhibitor mixture) and then washed twice with Tris-EDTA buffer (10 mM Tris-HCl, pH 7.9, 1 mM EDTA). The protein-DNA complexes were disrupted with proteinase K (Sigma-Aldrich) at pH 6.8. DNA was extracted with phenol and chloroform, precipitated in ethanol, and analyzed by real-time PCR using the Power SYBR^®^ Green Master Mix (Invitrogen Co.), and the primers are shown in Table 1.

### 2.8. Immunofluorescence

Cells were cultured in Iscove’s modified Dulbecco’s medium containing 1% BSA and 4% bovine serum at 37 °C. After *in situ* extraction with 0.1% Triton X-100 at 4 °C for 3 min, the cells were fixed in 4% paraformaldehyde for 20 min and permeabilized in PBS containing 0.1% saponin and 3% BSA at room temperature. Cells were stained with anti-JDP2 [9,10] and anti-Nrf2 or anti-MafK antibodies (all from Santa Cruz Inc.) for 2 h, washed with PBS containing 0.1% saponin, and stained with Alexa Fluor 488- or Alexa Fluor 546-conjugated secondary antibodies for 1 h. For DNA staining, cells were treated with 200 µg/mL RNase A for 30 min and 25 ng/mL TOPRO-3 for 30 min. The stained cells were mounted with anti-fade reagent. Confocal and Nomarski differential-interference contrast images were obtained using an FV500 laser-scanning microscope (Olympus, Tokyo, Japan). One-planar (xy) section slice images with a thickness of 2.0 μm were acquired. To ensure that there was no bleed through from the Alexa Fluor 488 signal into the red channel, Alexa Fluor 488 and Alexa Fluor 546 were excited independently at 488 nm and 543 nm, respectively. Emission signals were detected between 505 and 525 nm for Alexa Fluor 488, and between 560 and 600 nm for Alexa Fluor 543. TOPRO-3 was excited at 633 nm, and its emission signal was detected at a wavelength greater than 660 nm. Composite figures were prepared using Photoshop CS4 and Illustrator CS4 software (Adobe System Inc., San Jose, CA, USA). For staining of iPSCs, antibodies against JDP2, cyclin D1 (Calbiochem Billerica, MA, USA), SSEA1, SSEA3, SSEA4, TRA-1-60 and TRA1-81 (Millipore, Billerica, MA), OCT3/4, NANOG, SOX2, KLF4 and C-Myc (ABcam) were used. Cells were fixed with 4% formalin for 30 min in room temperature, and washed with PBS. 700 μL blocking solution (10% FBS, 1% Triton X-100, 0.1 M PBS) was added for 30 min, 700 μL of primary antibody solution was added, the cells were incubated overnight in 4 °C, and 700 μL of Alexa Fluor 488-conjugated secondary antibody (Invitrogen) solution was added for 1 h at room temperature. After washing in PBS, 700 μL of DAPI (Invitrogen; 0.1 μg/mL; diluted in PBS) was added for 10 min at room temperature, and the cells were stored at 4 °C until analyzed by fluorescence microscopy.

**Table 1:** Sequences of primers of ChIP assay.

Primer’s name	Sequences of primers	Product size
Mouse E1 NQO1	Forward: 5'-GCACGAATTCATTTCACACGAGG-3'	128 bp
	Reverse: 5'-GGAAGTCACCTTTGCACGCTAG-3'	
Mouse E1 HO-1	Forward: 5'-AGCGGCTGGAATGCTGAGT-3'	184 bp
	Reverse: 5'-CCTTCTGCCTGCCGTTCCGG-3'	
Mouse E2 HO-1	Forward: 5'-GGGCTAGCATGCGAAGTGAG-3'	201 bp
	Reverse: 5'-GACTCCGCCCCTAAGGGTTC-3'	

### 2.9. Reprograming and Generation of DAOY Medulloblastoma iPSC

Human *OCT3/4*, *SOX2*, *KLF4*, and *c-MYC* (human “four-factors”) cDNAs were amplified by reverse transcription-PCR (RT-PCR) using mRNA prepared from hES cells as the template with the primer sets as described previously [42]. The cDNAs were inserted into the pENTR/D-TOPO entry vector plasmid (Invitrogen) and verified by sequencing. The cDNAs in pENTR/D-TOPO were then transferred to the pCSII-EF-MCS-IRES2-Venus lentiviral vector plasmid using Gateway LR clonase (Invitrogen). Lentiviral vectors pseudotyped with the vesicular stomatitis virus G glycoprotein (VSV-G) were produced by transient transfection of three plasmids into 293T cells (ATCC): the packaging plasmid (pCAG-HIVgp), the vesicular stomatitis virus G glycoprotein- and Rev-expressing plasmid (pCMV-VSV-G-RSV-Rev), and the lentiviral vectorplasmid. The culture supernatant was concentrated by ultracentrifugation, and the viral pellet was resuspended in HBSS. The titers of vectors were determined by infection of HeLa CD4+ cells with serial dilutions of the vector stocks followed by fluorescence-activated cell sorting analysis for Venus+ cells. DAOY cells were cultured overnight in DMEM (Invitrogen) supplemented with 10% FBS-containing lentiviruses at a multiplicity of infection of 3.0 in flat-bottomed 24-well plates at 37 °C under 5% CO2 in air. Two days after transduction, the cells were harvested by trypsinization; 5 × 10^5^ cells were replated into 100-mm culture dishes and cultured on mitomycin C-treated mouse embryonic fibroblasts at a concentration of 5 × 10^6^ at 37 °C under 5% CO2 and 5% O2 in air. Hypoxic conditions were sustained for 14 days. The culture medium of iPSC (iPSM) [42] comprised 78% DMEM/Ham’s F-12 supplemented with 20% knock-out serum replacement (KSR, Invitrogen), 2 mM GlutaMax (Invitrogen), 1% nonessential amino acids, 0.1 mM β-mercaptoethanol, 10^3^ units/mL human LIF (leukemia inhibitory factor; Invitrogen), and 4 ng/mL human recombinant basic fibroblast growth factor (bFGF) (Wako, Osaka, Japan). On days 12–18, rabbit ESC-like cell colonies were isolated mechanically and replated onto mouse embryonic fibroblasts. iPSCs were passaged by incubating the cells with detaching solution (0.25% trypsin (w/v), 0.1 mg/mL collagenase IV, 20% KSR, 1 mM CaCl_2_) at room temperature and mechanically disaggregating the resulting small clumps into single cells. Cells were then counted in a hemocytometer, resuspended, and plated in iPSM supplemented with 8 ng/mL bFGF. Fresh medium was added daily and cells were passaged every 3 days. Colonies were selected for staining for alkaline phosphatase and stem cell markers, and analyzed further. Alkaline phosphatase staining was performed with the ES cell Characterization Kit (Chemicon Millipore) according to the manufacturer’s protocol.

### 2.10. RT-PCR and Quantitative PCR Analysis of iPSCs

Total RNA was isolated using ISOGEN (Nippon Gene, Toyama, Japan) from cells cultured under different conditions. After DNase treatment to prevent genomic DNA contamination, first-strand cDNA was synthesized using an RNA PCR kit (TaKaRa, Shiga, Japan) using an oligo (dT)-3' site adaptor primer. Synthesized cDNA was subjected to PCR using the specific primers listed in Table 2 using the following protocol: 94 °C for 3 min, 35 cycles at 94 °C for 30 s, 60 °C for 30 s, and 72 °C for 30 s. For quantitative PCR, RNA was transcribed to cDNA (100 ng/μL), and primer and 2× buffer (Qiagen SYBR Green) were added, and the PCR was performed for 35 cycles in a Roche ROCHE LightCycler 480. The primers are listed in Table 3.

### 2.11. Teratoma and Tumor Formation in SCID Mice

DAOY and 2F- and 4F-transfected cells (1 × 10^6^ cells) were resuspended in 140 uL prewarmed DMEM/F12 with 60 μL Matrigel (BD Bioscience, San Jones, CA, USA) and then injected subcutaneously to SCID mice to induce teratoma or tumor formation assay, which was analyzed using the protocol of the National Stem Cell Bank (NSCB Procedure SOP-CH-213 rev A). Mice were observed on a weekly basis. Tumor size was calculated as the product of width^2^ × length/2. The teratoma and cancers were fixed in 4% paraformaldehyde overnight and embedded in paraffin 6–8 weeks after injection. Sections were stained with hematoxylin and eosin.

### 2.12. Statistical Analyses

Differences between the treatment and the control conditions were identified using one-way analysis of variance and SPSS-16 Software (IBM Corp., Armonk, NY, USA). The data are presented as mean and standard error of the mean of 3–5 samples per assay. Comparisons were made using a two-tailed Student’s *t*-test for repeated measures. A *p* value < 0.05 was considered significant.

**Table 2:** Primer sequences for RT-PCR.

Gene	Sequence (5'→3')
****OCT4-F****	CCCCCTGTCTCCGTCACCAC
****OCT4-R****	CCACATAGCGTAAAAGGAGCA
**SOX2-F**	ACACTGCCCCTCTCACACATG
**SOX2-R**	CCACATAGCGTAAAAGGAGCA
**KLF4-F**	GACCACCTCGCCTTACACATG
**KLF4-R**	CCACATAGCGTAAAAGGAGCA
**c-MYC-F**	CAGCTACGGAACTCTTGTGC
**c-MYC-R**	CCACATAGCGTAAAAGGAGCA
**NANOG-F**	CAGTCTGGACACTGGCTGAA
**NANOG-R**	CTCGCTGATTAGGCTCCAAC
**NODAL-F**	GGGCAAGAGGCACCGTCGACATCA
**NODAL-R**	GGGACTCGGTGGGGCTGGTAACGTTTC
**DPPA2-F**	CCGTCCCCGCAATCTCCTTCCATC
**DPPA2-R**	ATGATGCCAACATGGCTCCCGGTG
**DPPA4-F**	GGAGCCGCCTGCCCTGGAAAATTC
**DPPA4-R**	TTTTTCCTGATATTCTATTCCCAT
**hTERT-F**	CCTGCTCAAGCTGACTCGACACCGTG
**hTERT-R**	GGAAAAGCTGGCCCTGGGGTGGAGC
**EBAF-F**	GCTGGAGCTGCACACCCTGGACCTCAG
**EBAF-R**	GGGCAGCGAGGCAGTCTCCGAGGC
**ESG1-F**	ATATCCCGCCGTGGGTGAAAGTTC
**ESG1-R**	ACTCAGCCATGGACTGGAGCATCC
**DNMT3 *β*-F**	TGCTGCTCACAGGGCCCGATACTTC
**DNMT3 *β*-R**	TCCTTTCGAGCTCAGTGCACCACAAAAC
**UTF-1-F**	CCGTCGCTGAACACCGCCCTGCTG
**UTF-1-R**	CGCGCTGCCCAGAATGAAGCCCAC
**GAPDH-F**	GACTCCGACCTTCACCTTC
**GAPDH-R**	GAAATCCCATCACCATCTTC

**Table 3:** Primer sequences for qPCR.

Gene	Sequence (5'→3')
**OCT4-F**	GGGTTTTTGGGATTAAGTTCTTCA
**OCT4-R**	GCCCCCACCCTTTGTGTT
**SOX2-F**	CAAAAATGGCCATGCAGGTT
**SOX2-R**	AGTTGGGATCGAACAAAAGCTATT
**KLF4-F**	AGCCTAAATGATGGTGCTTGGT
**KLF4-R**	TTGAAAACTTTGGCTTCCTTGTT
**c-MYC-F**	CGGGCGGGCATTTG
**c-MYC-R**	GGAGAGTCGCGTCCTTGCT
**NANOG-F**	CAGTCTGGACACTGGCTAA
**NANOG-R**	CTCGCTGATTAGGCTCCAAC
**GAPDH-F**	GAAGGTGAAGGTCGGAGTC
**GAPDH-R**	GAAGATGGTGATGGGATTTC

## 3. Results

### 3.1. JDP2 Reduces H_2_O_2_-Mediated Production of ROS and Increases Antioxidant Activity

**Figure 1:**
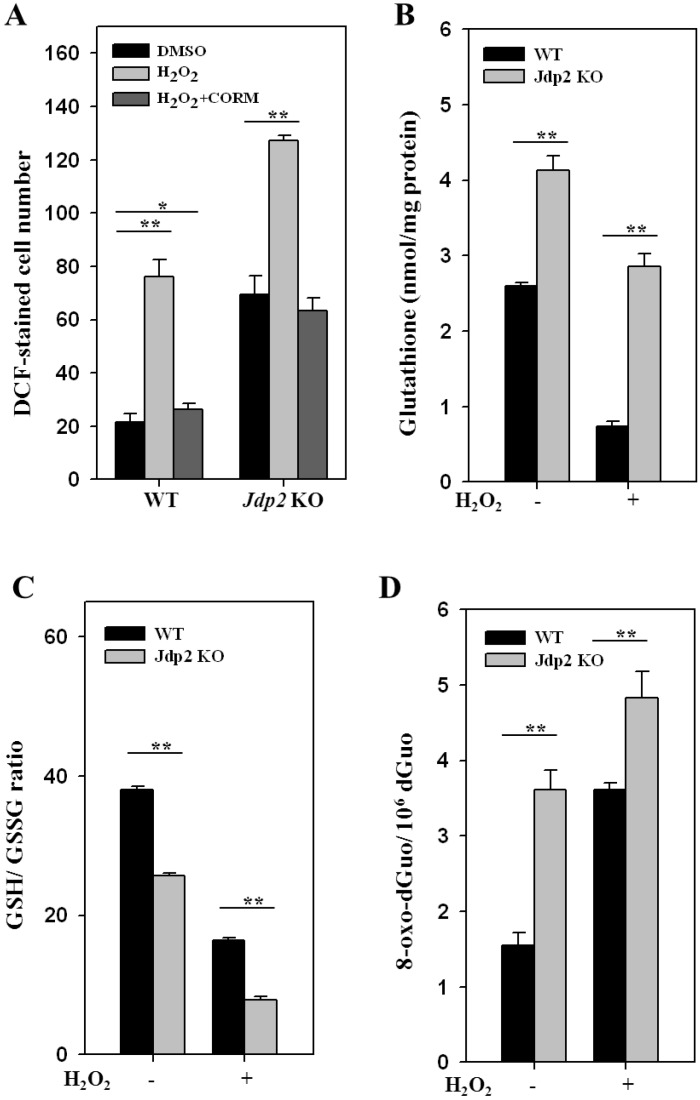
JDP2 controls ROS production. (**A**) MEFs were seeded (5 × 10^3^ per well) into eight-chambered cover glasses immersed in 500 μL of 10% FBS-DMEM and exposed to H_2_O_2_ (5 × 10^–6^ M) alone or with 50 nmol/L of CORM for 3 h, with medium changes every 30 min. Intracellular ROS levels were measured using H_2_DCFDA staining, as described in the Materials and Methods. (**B**) Level of glutathione in WT and *Jdp2* KO MEFs incubated with H_2_O_2_ (5 × 10^–6^ M). (**C**) Determination of the GSH/GSSG ratio in WT and *Jdp2* KO MEFs incubated with H_2_O_2_ (5 × 10^–6^ M). (**D**) 8-oxo-dGuo levels in WT and *Jdp2* KO MEFs in the presence or absence of H_2_O_2_ (5 × 10^–6^ M). Data are presented as mean ± SD. The data were analyzed using Student’s *t* test. **, *p* < 0.01.

To study the role of JDP2 in the induction of ROS, MEFs from WT and *Jdp2* KO mice were exposed to H_2_O_2_, a ROS-generating agent. ROS production was 3.2-fold higher in *Jdp2* KO MEFs than in WT MEFs in the absence H_2_O_2_. Addition of H_2_O_2_ increased ROS production by about 3.8-fold in WT MEFs and 1.9-fold in *Jdp2* KO MEFs. CORM, an ROS inhibitor, abolished these increases in ROS production (Figure 1A). Thus, we conclude that JDP2 inhibits the H_2_O_2_-mediated production of ROS.

Because ROS alterations can affect the intracellular redox state [40], the ratio of reduced to oxidized glutathione (GSH/GSSG) was also examined in WT and *Jdp2* KO MEFs in the presence or absence of H_2_O_2_. The level of total glutathione was enhanced 1.7-fold in *Jdp2* KO MEFs compared with WT MEFs in the absence of H_2_O_2_, and 4.0-fold in the presence of H_2_O_2_, respectively (Figure 1B); however, the GSH/GSSG ratio in *Jdp2* KO MEFs was reduced to 67.5% of that seen WT MEFs in the absence of H_2_O_2_, and to 53% in the presence of H_2_O_2_, thus indicating a more oxidized intracellular environment in *Jdp2* KO MEFs (Figure 1C). Addition of H_2_O_2_ reduced GSH/GSSG value to 32% in *Jdp2* KO MEF and to 40.5% in WT MEFs relative to those in the absence of H_2_O_2_. Moreover, the level of 7,8-dihydro-8-oxo-2'-deoxyguanosine (8-oxo-dGuo), which is one of the major products of DNA oxidation, increased 2.4-fold in *Jdp2* KO MEFs compared with WT MEFs in the absence of H_2_O_2_, and H_2_O_2_ induced the production of 8-oxo-dGuo 1.3-fold in *Jdp2* KO MEFs compared with WT MEFs (Figure 1D). Thus, the levels of ROS, total glutathione, and 8-oxo-dGuo were increased, whereas the GSH/GSSG ratio was decreased, in *Jdp2* KO MEFs compared with WT MEFs in the presence or absence of H_2_O_2_. These results support the hypothesis that these differences result from differential activation of ROS production in *Jdp2* KO MEFs as compared with WT MEFs.

### 3.2. JDP2 Is Involved in ARE Transactivation

**Figure 2:**
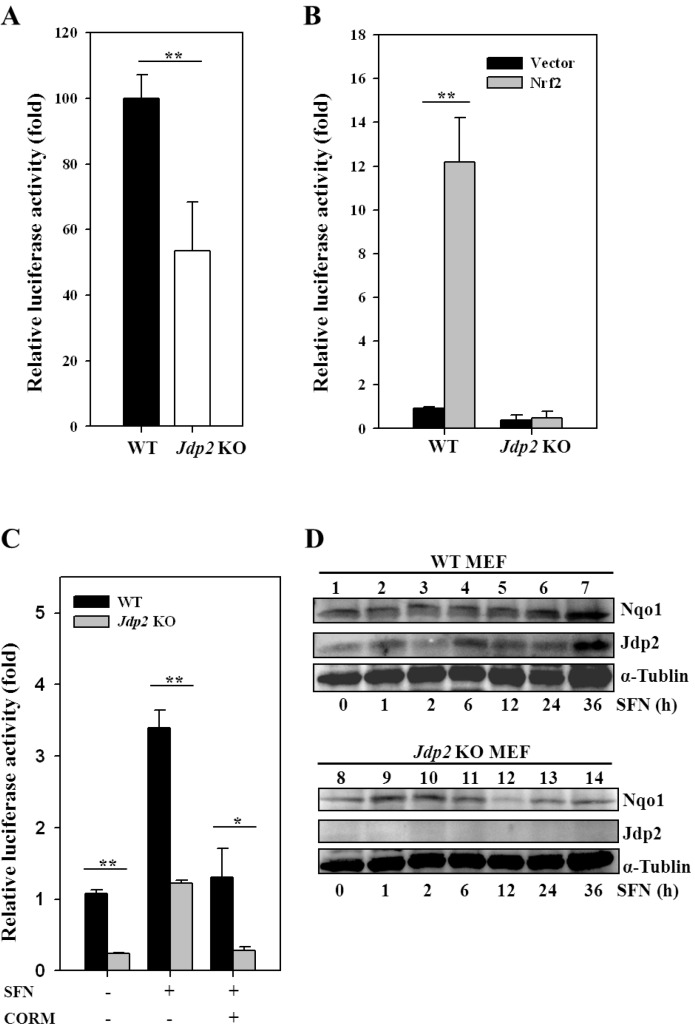
JDP2 is involved in SFN-induced ARE transactivation. (**A**) Relative *NQO1* promoter activity in WT and *Jdp2* KO MEFs. Transfectants with pGL4-hQR25-luciferase were incubated further for 24 h after transfection, and luciferase activity was measured as described in Materials and Methods. (**B**) Effect of JDP2 on ARE activity in the presence of Nrf2. WT and *Jdp2* KO MEFs (5 × 10^4^) were transfected with 400 ng of pGL4-hQR25-luciferase, 50 ng of pcDNA3-Nrf2, and the indicated amounts of pcDNA-JDP2. One day after transfection, cells were harvested and luciferase activity was measured. **, *p* < 0.01. (**C**) Effect of CORM on *NQO1* promoter activity. WT and *Jdp2* KO MEFs were exposed to SFN (5 × 10^–6^ M) alone or in the presence of 50 nM/L of CORM. After 24 h of culture, luciferase activity was measured. Each value represents the mean ± SD (n = 3). *, *p* < 0.05; **, *p* < 0.01. (**D**) Proteindetection in WT and *Jdp2* KO MEFs treated with SFN. After 24 h of culture, SFN (5 × 10^–6^ M) was added and cells were cultured for an additional 12 h. Cellular lysates from WT and *Jdp2* KO MEFs (40 µg) were separated on SDS-PAGE and transferred onto membranes, and the antibody specific for each protein of interest was used for immunodetection.

We next examined the H_2_O_2_-mediated induction of transcription via ARE [43]. In *Jdp2* KO MEFs, ARE-luciferase activity, as measured using the pGL4-hQR25 luciferase reporter plasmid, was reduced to about half the level observed in WT MEFs (Figure 2A). Furthermore the transactivation of Nrf2 was evident: a >12-fold Nrf2-mediated induction was detected in WT MEFs, whereas only little increase was found in *Jdp2* KO MEFs (Figure 2B), suggesting that the presence of JDP2 in the Nrf2-ARE complex is critical for ARE activity.

SFN is a potent inducer of phase II detoxification enzymes and inhibits tumorigenesis in animal models [44]. To determine whether JDP2 is involved in SFN-induced ARE transactivation, we transfected an ARE-luciferase plasmid into both WT and *Jdp2* KO MEFs and treated the cells for 24 h with 5 × 10^−6^ M SFN (Figure 2C). The SFN-induced increase in ARE was maximal at 24 h after exposure to SFN (Supplementary Figure S1). In the absence of SNF, the ARE activity in *Jdp2* KO MEFs was reduced to about 30% relative to that in WT MEFs. SNF increased ARE transcription to 3.4-fold in WT MEFs and 4.0-fold in *Jdp2* KO MEFs; however, both activities were reduced to the original level in the presence of CORM. The expression of NQO1 protein increased by 2.5-fold at 24–36 h after incubation with SFN in WT MEFs but not in *Jdp2* KO MEFs (Figure 2D). The expression of JDP2 was also increased by 3.5-fold at 36 h after incubation with SFN in WT MEFs.

### 3.3. Colocalization and Recruitment of Nrf2, MafK, and JDP2

**Figure 3:**
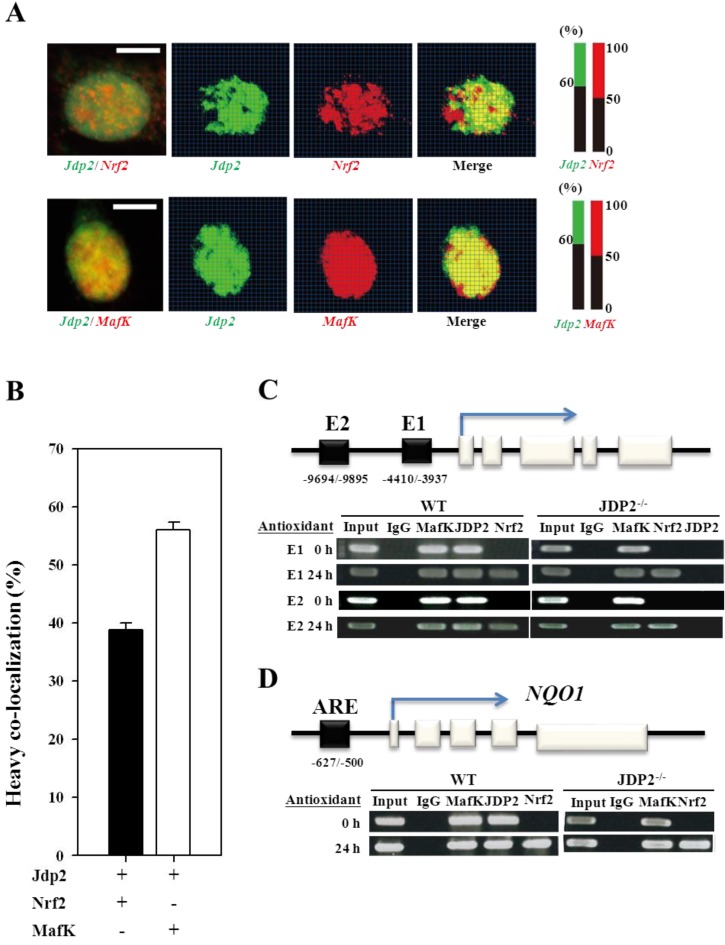
Colocalization and recruitment of JDP2 with Nrf2 or MafK. HeLa cells were triply stained with anti-JDP2, anti-Nrf2 (JDP2-Nrf2), or anti-MafK (JDP2-MafK) antibodies and with TOPRO-3. (**A**) The quantitation of colocalization was evaluated with the intensities of green, red and the yellow as merged dots. Respective intensity was subdivided into small square areas and calculated their total intensity relative to the intensity of DAPI. Scale bars, 10 µm. (**B**) Quantitation of the merged ration between JDP2 and Nrf2, or JDP2 and MafK. The values were the mean values of three in dependent experiments. (**C**,**D**) ChIP assay of Nrf2, MafK, and JDP2 with the ARE. TPA enhances the recruitment of Nrf2 to the ARE of the *HO-1* (**C**) and *NQO1* (**D**) genes. Extracts from TPA-treated (24 h) or nontreated MEFs (5 × 10^8^ cells) from WT and *Jdp2* KO mice were subjected to ChIP assays with indicated antibodies. Precipitated DNA and input DNA (1/20-fold) were analyzed using PCR with primers that were specific for E1 and E2 of the *HO-1* and E1 of the *NQO1* promoters as described in Experimental section.

To confirm the colocalization of Nrf2, MafK, and JDP2, we used Alexa Fluor 488 and Alexa Fluor 546 as secondary antibodies. Signals corresponding to Nrf2 localized in the regions of decondensed chromatin and appeared mainly as nuclear foci (Figure 3A). By contrast, the signals of JDP2 and MafK were detected in all regions of the nuclei. Thirty-eight percent of Nrf2 and 56% MafK colocalized with JDP2 (Figure 3B). Thus, the complex encompassing Nrf2, MafK, and JDP2 seems to be present in the nuclei of HeLa cells. This colocalization was also confirmed using a ChIP assay, as described below.

### 3.4. Nrf2, MafK, and JDP2 Proteins Are Recruited to the ARE

The complex formed by Nrf2, MafK, and JDP2 was examined in WT and *Jdp2* KO MEFs to understand their recruitment to the ARE of *HO-1* and *NQO1* genes, which belong to the antioxidative stress enzyme family [3,6,45]. We designed the locus of the AREs at E1 and E2 sites, which contain multiple stress-responsive elements [46] and also conform generally to the Maf-recognition element [47]. Jdp2 and MafK were recruited to the E1 and E2 sites in WT MEFs, but only MafK was recruited to the E1 and E2 sites in *Jdp2* KO MEFs (Figure 3C). In response to the antioxidant TPA, the recruitment of Nrf2, Jdp2, and MafK was significant in WT MEFs; however, only Nrf2 and MafK were recruited in *Jdp2* KO MEFs. Thus, the E1 and E2 sites may be critical for the differential regulation of Nrf2, MafK, and Jdp2 on the *HO-1* enhancer, and JDP2 seems to be the key regulator of Mrf2/MafK recruitment and the ARE response. Similar results were observed for the ARE of the *NQO1* gene (Figure 3D). Jdp2 was recruited to ARE of the *NQO1* gene together with Nrf2 and MafK proteins in response to the antioxidant in WT MEFs.

### 3.5. Expression of Jdp2 Is Restricted in the Cerebellum

The targeting vector contained a 2.5 Kb DNA fragment 5' of the transcription initiation site of *Jdp2* and a Cre minigene with IRES and NLS followed by a SV40 polyadenylation signal (Supplementary Figure S2A). Transgenic mouse lines (F_0_) were obtained and crossed with ROSA26R reporter mice [48] and Z/EG mice [49]. Functional F_1_ offspring from each transgenic line were identified by X-gal staining, GFP signaling and genotyping by PCR. Sibling breeding of double transgenic mice of *Jdp2*-Cre/ZEG or *Jdp2*-Cre/ROSA26R was used to increase the colony size of the double-transgenic mice. Surprisingly, animals derived from *Jdp2*-Cre/ZEG mice expressed green fluorescence predominantly in the cerebellum (Supplementary Figure S2B,C). Similar staining of X-gal was observed as for *Jdp2*-Cre/ROSA26R. RT-PCR was performed with specific primers for the transcripts of *Jdp2* to verify whether the observed promiscuous Cre activities were derived from transcription activities of such cerebellum-specific genes during gametogenesis because of the leaky transcription of the *Cre*-minigene driven by the tissues-specific promoters. *Jdp2*-specific transcript was detected in the brain and lung [11]. GFP expression was also examined in the brain of *Jdp2*-Cre/ZEG mice, especially in the cerebellum. The GFP signals were detected in the cerebellum granule cells in sagittal sections of the brain (Supplemental Figure S2D). X-gal staining of *Jdp2*–Cre/ROSA26R showed that signals localized in the cerebellar granule cells, which confirmed the finding for anti-GFP staining of the brain in *Jdp2*-Cre/ZEG mice. These data indicate that the JDP2 is expressed in the cerebellum, particularly in the granule cells.

### 3.6. JDP2 and OCT4 Cause Nuclear Reprogramming of Medduloblastoma Cells

JDP2 plays a critical role in cell cycle arrest through regulated expression of cyclin A2 and p16^Ink4a^, which are involved with the p19^Arf^-Mdm2-p53-p21^Cip1^-cyclin/cyclin-dependent kinase (CDK) and p16^Ink4a^-cyclin/CDK-Rb-E2F networks [11]. The forced expression of JDP2 increased the expression of p16^Ink4a^ and p19^Arf^ [17]. WNT/GSK3β signaling to estrogen-related receptor beta (Esrrb) is also critical for generating and maintaining the stemness characteristics of iPSCs [27,34]. Previously, we showed that the expression of JDP2 is reduced significantly in hypoxia compared with normaxia [17], and JDP2 regulates the expression of Wnt targets including LEF1/TCF [11].

Nuclear reprogramming involves extensive chromatin remodeling and resets epigenetic program to generate iPSCs [50]. Increased ROS by metabolic changes in iPSCs may hinder survival of reprogrammed cells, as suggested by observations of iPSC-generation under hypoxia condition [34,35]. In addition, mitochondrial contents are also repressed in iPSCs or hESCs [36], suggesting that ROS generation by reprogramming factors is unfavorable to iPSCs. Thus, introduction of JDP2 may be useful to trigger the iPSC-generation because JDP2 inhibits the ROS production.

We also know that the main target of gene-expression of JDP2 is restricted in the brain cerebellum granule cells, as described above. Thus, we focused on the medduloblastoma to generate the iPSCs through the combination of JDP2 and defining factor OCT4.

**Figure 4:**
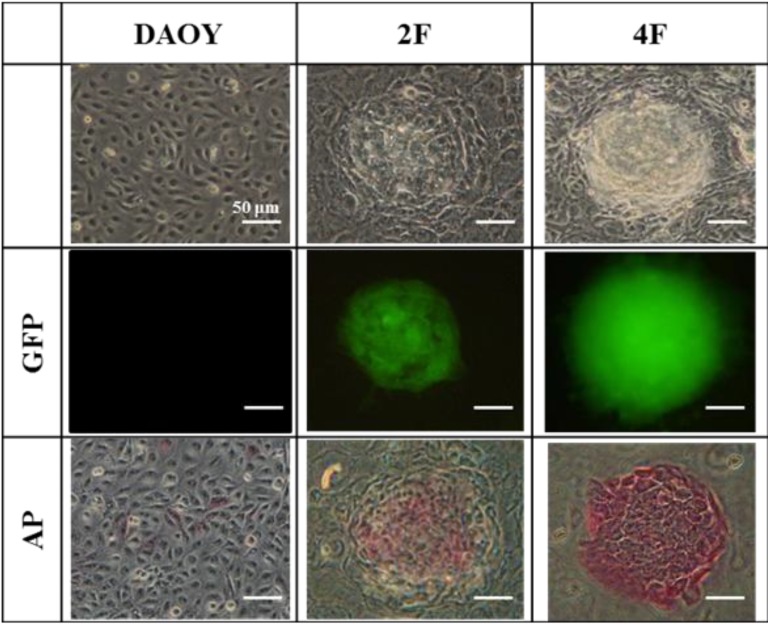
Generation of two-factor OCT4-JDP2 (2F-) iPSC-like cells and conventional four-factor OCT4-SOX2-KLF4-c-MYC (4F-) iPSC-like cells from DAOY medulloblastomacells. Morphology of DAOY, 2F and 4F iPSC-like cells and alkaline phosphatase activity (AP) are shown. Phase contrast picture and AP staining were shown. Scale bar; 50 μm.

**Figure 5:**
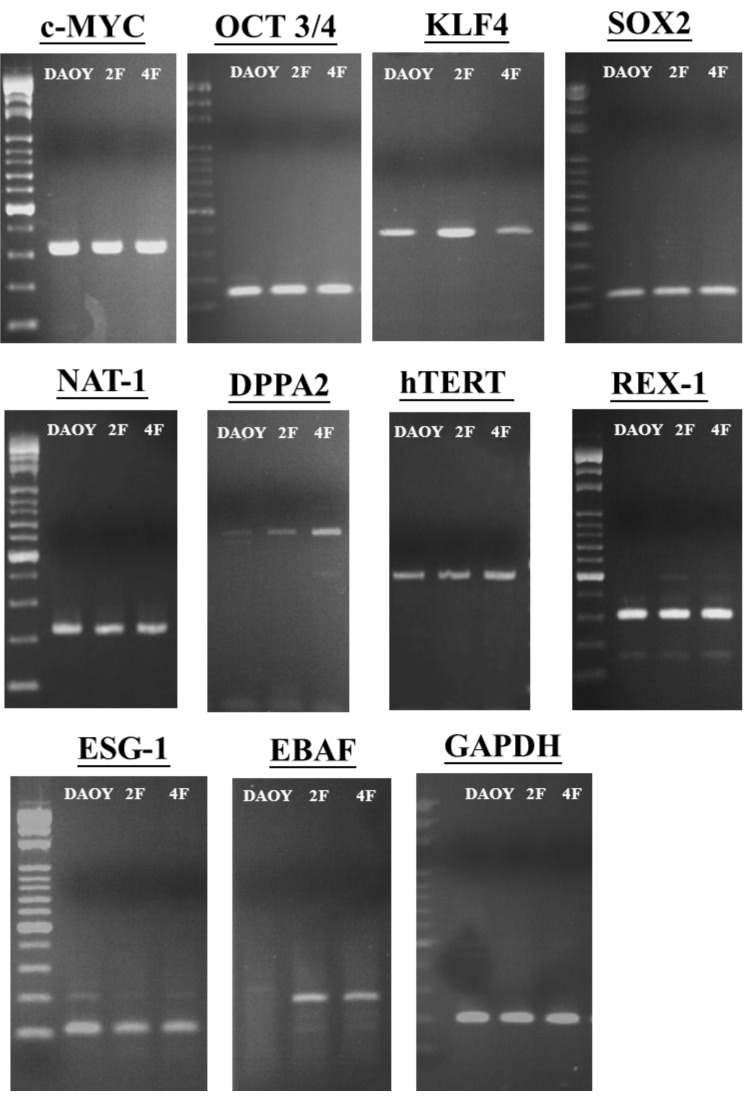
Expression of stemness marker genes in DAOY, 2F- and 4F-iPSC-like cells. RT-PCRanalysis of stemness marker genes. A series of stemness genes and GAPDH as a positive amplification and loading control are shown. The sequences of specific primers were listed in Table 2 and followed to the protocol of RT-PCR as described in Experimental Section.

In the human DAOY cells, we detected endogenous expression of OCT4, SOX2, KLF4, C-MYC, and NANOG. Ｍost stemness-genes were expressed in DAOY cells, however, no or weak band of JDP2 was detected by Western blotting using monoclonal antibody against JDP2, #176 (Supplementary Figure S3), and DAOY cells was not stained by alkaline phosphatase (Figure 4, Figure 5). Thus, DAOY is not the stem cells. Therefore, we predicted that OCT4 itself is capable of reprogramming the DAOY medulloblastoma cells via a network leading to the expression of OCT4, SOX2, and NANOG. However, introduction of only OCT4 did not generate iPSC-like cells from DAOY cells (data not shown). Thus, we next examined two-factor such as JDP2 and OCT4 to induce iPSCs (2F-iPSCs) because JDP2 regulates WNT signaling pathway and prevent ROS production [11]. We also tried adding the defined four factors OCT4, SOX2, KLF4, and C-MYC to try to generate iPSC-like cells form DAOY cells (4F-iPSCs). Small, packed, domed colonies were detected on mitotically inactivated MEF cells 17 days after lentivirus transduction. These colonies comprised small and rapidly dividing cells with a high nuclear-cytoplasmic ratio and large nucleoli. The estimated reprogramming efficiency was 0.3%, which was 20 times higher than the efficiency of the one-factor approach for reprogramming murine neural stem cells [51]. After colonies were selected manually, the iPSC-like cells were passaged. The number of colonies with the typical iPSC phenotype increased over time and with repeated passages. From the initial input of 5 × 10^4^ cells, we obtained 10 colonies at the second passage, from which we eventually obtained about 20 iPSC-like cell colonies. When iPSC-like cells were subcultured for more than 4 weeks under conditions specific for iPSC-like cells, the cells displayed strong alkaline phosphatase activity (Figure 4). Immunofluorescence staining confirmed the expression of the endogenous “stemness” markers, including SSEA3, SSEA4 and Tra-1-60 in the DAOY iPSC-like cells, but not in the DAOY original cells (data not shown). In 2F-iPSC-like cells, each staining signal was not strong compared with 4F-iPSC-like cells. Thus, 2F-iPSC-like cells seem to be incomplete although they exhibited the alkaline phosphatase activity. These markers were most intense in the dense patches of cells with typical iPSC morphology (data not shown). We also examined the expressions of stemness genes by RT-PCR analysis (Figure 5). Other *DPPA2*, *hTERT*, *NAT-1*, *REX-1*, *ESG*, and *GRB7* in iPSC-like cells showed the similar expression patterns of DAOY cells. The expression of *EBAF* was not detected in DAOY cells but detected in both 2F-iPSC-like cells and 4F-iPSC-like cells.

**Figure 6:**
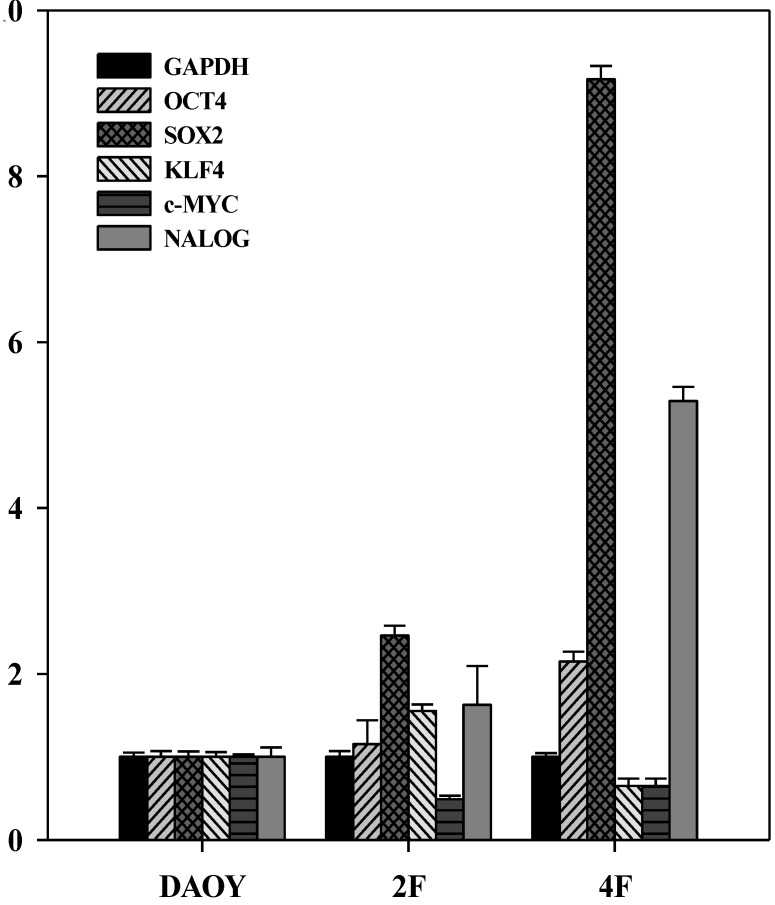
Gene expression of 2F- and 4F-iPSC-like cells. Quantitative real time PCR assay for expression of OCT4, SOX2, KLF4, and NANOG in human DAOY, 2F-and 4F-iPSC-like cells. Individual PCR reactions were normalized against internal control (GAPDH) and plotted relative to the expression level in DAOY. The sequences of the primers were shown in Table 3.

Transcriptions of *OCT4*, *SOX2*, *c-MYC*, and *KLF4* were also detected and quantitated their expressions in the iPSC-like cells (Figure 6). In particular, the expression of *SOX2* was significantly higher than other stemness genes in 2F- and 4F-iPSC-like cells. In 4F-iPSC-like cells, the oncogenes such as *C-MYC* and *KLF4* were not expressed higher, instead the stemness genes such as *SOX2* and *NANOG* expressed at significant levels (5.3–9.1-fold, respectively). By contrast, 2F-iPSC-like cells only showed higher expression of *SOX2*. In both iPSC-like cells, expression of oncogenic *c-MYC* was repressed significantly.

### 3.7. iPSCs Exhibit Pluripotency

Human ESCs will form teratoma-like masses after cell injection into immunodeficient mice, an assay that has become the accepted standard for demonstrating their developmental pluripotency [34]. Thus, to assess the pluripotency of the iPSC-like cells *in vivo*, we transferred the cells into immunodeficient SCID mice [52]. The DAOY generated benign cystic teratomas containing mature tissues from endoderm and ectoderm, but not mesoderm layers. However, sometimes the neoplasia was found in the tissue derived from neural ectoderm in 2F- or 4F-iPSC-like cells (data not shown).

### 3.8. Tumor Formation

**Figure 7:**
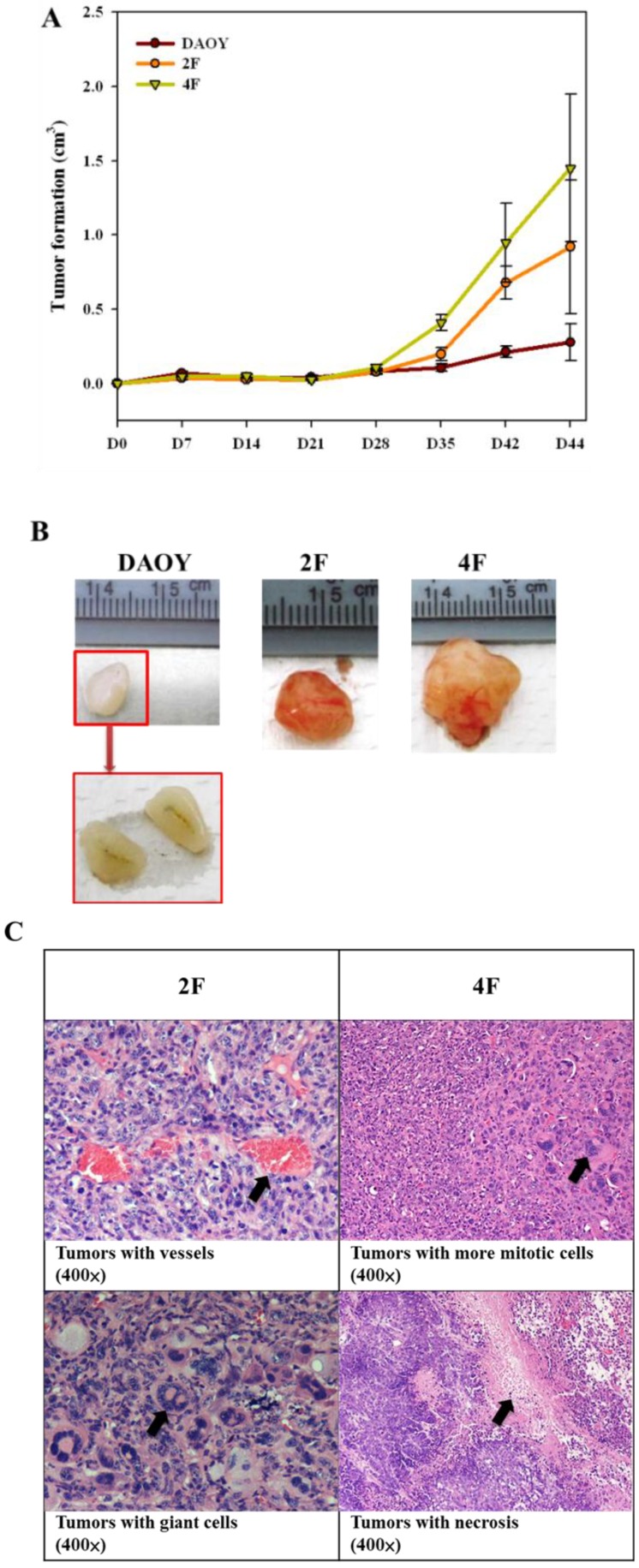
Tumor formation of DAOY, 2F- or 4F-iPSC-like cells in SCID mice. (**A**) Time course of tumor sizes in the SCID mice with subcutaneously injected with DAOY, 2F-iPSC-like cells (2F), and 4F-iPSC-like cells (4F). Tumor sizes were measured as indicated in Experimental Sections. The tumor sizes were getting bigger on 28 days after transplantation. (**B**) Tumors of DAOY (left), 2F-iPSC-like cells, 4F-iPSC-like cells were shown (right). Scale bars showed the sizes of tumors. (**C**) Tumors were appeared after day 44 after transplantation (×400 fold). 2F-iPSC-like cells generated malignant tumors with more vessels and giant cells (see arrow), and 4F-iPSC-like cells exhibited more malignant tumors with rapid mitosis and necrosis (see arrows). The black arrows indicted the possible malignant sites.

We injected the human iPSC-like cells into SCID mice. Tumor formation was repressed during the initial stage up to 28–35 days after injection; however, beginning 1 month after injection, significant growth occurred, and the tumor size increased by 2.5–3.5-fold (Figure 7A,B). We detected no signs of differentiation but found malignant growth of vessels, giant cell formation, and mitosis in sometimes in 2F- or 4F-iPSC-like cells (Figure 7C). Thus, we found that the teratoma sometimes generated the malignant tumors.

## 4. Discussion

In this study, we demonstrated the role of JDP2 in oxidative stress and ROS homeostasis, and the possible link between ROS homeostasis and nuclear reprogramming. During reprogramming, Yamanaka factors induced the chromatin changes to generate the stress response including ROS production [50]. The increasing of ROS inhibited the survival of reprogramming cells which led to DNA damages, cellular senescence and apoptosis [7]. Thus, the factor which regulates ROS homeostasis may play a critical role for efficient reprogramming [7]. We showed that ROS production is higher in *Jdp2* KO MEFs than in WT MEFs and that addition of an oxidative stress inducing reagent increased ROS production in *Jdp2* KO MEFs compared with WT KO MEFs (Figure 1). Conversely, ARE-luciferase activity increased more in WT MEFs than in *Jdp2* KO MEFs (Figure 1, Figure 2A). These data indicate that JDP2 is a critical factor for inhibiting ROS production and DNA oxidation. In addition, it was reported that senescence impaired the reprogramming to iPSCs, and reprogramming triggered a stress response of senescence at the initial stage [53]. In fact, senescence is the irreversible arrest during the G1 transition of the cell cycle elicited by replicative exhaustion or in response to stresses such as DNA damage, drugs, or oncogenes. This arrest is implemented primary through activation of p53 and the up-regulation of the cycling dependent kinase (CDK) inhibitors p16^Ink4a^ and p21^Cip1^ [54]. Introduction of Yamanaka factors triggers senescence by up-regulating p53, p16^Ink4a^, and p21^Cip1^ at initial stage, and at later stage these expressions were repressed. This reprogramming-induced senescence (RIS) acts as an initial barrier limiting the efficiency of the reprogramming. The reprograming is slower and stochastic, suggesting the existence of barrier limiting its efficiency. In order to increase the efficiency of the reprogramming, the repression of RIS is definitely required for full reprogramming. Overexpression of JDP2 increases the expression of p16^Ink4a^ and p19^Arf^, thus generates the replicative senescence through the repression of H3K27me3 by polycomb complex [17]. However, the reduced expression by shRNA against p16^Ink4a^ (Supplementary Figure S4), p21^Cip1^ or p53, increased nuclear reprogramming to generate iPSCs at pre-iPC stage. Thus, JDP2 is assumed to be a possible gene to induce this RIS at the initial stage during the commitment to iPSCs.

Another merits of JDP2 as one of the candidates reprogamming factor is that JDP2 connects with WNT signaling. The recent report showed that oxygen is known to regulate stem cells through WNT signaling and HIF1α [55]. Moreover, the GSK3β-TCF3 axis in WNT signaling controls self-renewal of stem cells [56]. We previously demonstrated; (I) JDP2 regulates WNT signaling [11], and (II) the expression of JDP2 is repressed under hypoxia conditions [17] and JDP2 inhibits the ROS production. Thus, we hypothesize that JDP2 controls nuclear reprogramming with combination of OCT4. The transcription factor OCT4 interacts physically with various active and repressive chromatin complexes; raising the question whether OCT4 or another reprogramming factor is more important for reprogramming [57]. It has been reported that high levels of OCT4 and low levels of SOX2 increase the efficiency of reprogramming [58,59]. We tried the combination of OCT4 and JDP2 to induce reprogramming. In this study, we replaced SOX2, and KLF4 and c-MYC by JDP2 in the alternative set of transcription factors for reprogramming, in addition to the traditional four-factor reprogramming. We studied the characteristics of the reprogrammed medulloblastoma cells because JDP2 was expressed predominantly in the cerebellar granule cells (Supplementary Figure S2).

Nanog is essential for the establishment of iPSCs and is expressed before many other pluripotency genes during the reprogramming process, suggesting that it may be required for their activation [60]. Moreover, the interplay of Nanog and JDP2 was reported in P19 cells [61]. Overexpression of NANOG was found in our iPSC-like cells (Figure 6), reflecting the maintenance of the self-renewal and stemness-charactristics in iPSCs-like cells. The appearance of positive stem cell markers such as alkaline phosphatase, OCT4, SOX2, and NANOG in these cells indicates pluripotency. However, our teratoma study showed that only two germ layers formed after subcutaneous injection of either type of transfected DAOY cells into SCID mice, which are similar in DAOY cells. In conclusion, we cannot generate the iPSCs from DAOY meddullobastoma cells, instead we generated the iPCCs which have higher potency to generate the cancer-inducing ability than the original DAOY cells by JDP2 and OCT4. Moreover when we introduced four factors, we failed to generate the complete iPSCs from DAOY cells.

Cancer cells exhibit several characteristics that also appear in ESCs or iPSCs [33]. Genomic instability, which leads to numerical and structural aberrations, is observed frequently in cancer and cultured pluripotent stem cells. These findings imply that iPSCs or iPSC-derived cells are potentially carcinogenic. Several human gastrointestinal cancer cell lines have been reprogrammed into iPCCs sharing the characteristic features with ESCs by overexpressing the established Yamanaka factors OCT4, KLF4, SOX2, and c-MYC [31]. Reprogramming of cancer cells *in vitro* may reflect a more complex mechanism than the reprogramming of normal cells because of aneuploidy, deregulation of signaling pathways, and the high proliferation rate of cancer cells. Moreover, iPCCs carry the burden of their cancer cell descent, requiring more work for the maintenance of iPCCs because cells differentiated from iPCCs can remain immortal and overgrow in cell culture [33].

Our data showed a more aggressive phenotype after 28 days in the DAOY iPCCs inoculated into SCID mice compared with the untransfected DAOY cells they were derived from. The questions arising from this observation are: to what extent does reprogramming alter cancer cell tumorigenicity to obtain the stemness characteristics; how the resistance capacity of ROS and the antioxidation ability of defined factors contribute to the generation of full reprogramming or the stability of genome; and which transcription factors to regulate the ROS homeostasis contribute to the differences in cell characteristics from those in iPSCs reprogrammed from somatic cells; and does reprogramming and ROS regulation in stem cells offer new understanding of cancer cell tumorigenicity or possibilities for cancer treatment?

Our data demonstarted that 2F such as JDP2 and OCT4 generated the cancer stem-like cells with stemness-characteristics. How does JDP2 induce the cancer phenotype during reprogramming? One possible explanation is the mutation of *p53* gene. JDP2 regulates the expression of *p53* gene [62]. We reported that *Jdp2* KO MEFs showed the reduced expression of p53 and p21^Cip1^ [11]. However, others reported the contradicted results [21]. Moreover, Aronheim *et al.* [22] demonstrated that the initial expression of JDP2 is critical for tumor suppressor function, after which it potentiates hepatocellular carcinoma with higher mortality and increased number and size of tumors in mice. However, we do not know whether the reprogramming could shut down the expression of this mutant p53 in DAOY-iPSCs or maintain its mutation of *p53* gene, even though the epigenomes are resetted. During the reprogramming, the new mutation of p53 might also be arisen in the genome of DAOY iPSCs. Assuming p53 remains to be mutated in iPSCs, it could be easily produce the cancer when it meets the second hit. We need further study to clarify the mechanism how the DAOY-iPSC-like cells generate tumors.

Miyoshi *et al*. reported a lower tumorigenic potential and increased the sensitivity to 5-fluorodeoxyuridine compared with the untransfected cancer cell line after subcutaneous transplantation of post-iPCCs into the dorsal flank of immunodeficient mice [32]. Similarly, Carette *et al*. found that reprogrammed iPCCs from chronic myeloid leukemia cells developed resistance to treatment [31]. Decreased susceptibility might explain why some chemotherapeutics or targeted therapies cannot cure certain types of cancers fully, assuming that putative cancer stem cells or defined populations of cells within the tumor exhibit a less differentiated state. Thus, the further study is required for complete understanding of the pluripotency, self-renewal, gene expression, epigenetic changes, mutation of tumor suppressor genes, and immune surveillance among iPSCs, ESCs and CSCs.

Hopefully, this approach will help to identify new tumorigenesis-related genes or epigenetic changes that can be explored for the development of new anticancer drugs, which targets on the control of ROS generation. Because iPCCs can differentiate into cancer cells or may acquire more carcinogenic characteristics after reprogramming, development of suitable model for studying tumorigenesis *in vitro* is necessary. Reprogramming of cancer cells and the regulation of ROS are an exciting new approach for basic and therapeutic research and might offer new possibilities in future.

## 5. Conclusions

JDP2 acts as an AP-1 repressor protein to suppress cell proliferation during cancer progression and participates in the maintenance of ROS homeostasis to prevent cell damage by ROS. *Jdp2*-Cre mice exhibited restricted expression in the granule cells of brain cerebellum. We generated iPSC-like cells form DAOY medulloblastoma cell by introducing JDP2 and the defining factor OCT4. iPSC-like cells expressed stem cell-like characteristics including alkaline phosphatase and some stem cell markers. However, such iPSC-like cells also proliferated rapidly, formed neoplasias in SCID mice, and potentiated cell malignancy. This study showed that the antioxidant-induced transcription factor JDP2 modulates ROS activity and that addition of JDP2 and OCT4 led to reprogramming of DAOY medulloblastoma cell to iPSC-like cells. However these iPSC-like cells caused cancer although they showed stemness characteristics, indicating that JDP2 controls the nature of iPSCs and their ability to develop into cancer stem cell-like cells. This will be helpful for studying the generation of cancer stem cells and ROS homeostasis.

## Supplementary Files

Supplementary File 1 Supplemental Materials (PDF, 303 KB)
