# Supplementary material for: Control of Oxidative Stress and Generation of Induced Pluripotent Stem Cell-like Cells by Jun Dimerization Protein 2
Source: Cancers (Basel). 2013 Jul 26;5(3):959–84. doi: 10.3390/cancers5030959 (PMC3795374; doi:10.3390/cancers5030959)
Supplement: Supplementary File 1 — Supplemental Materials (PDF, 303 KB) [file cancers-05-00959-s001.pdf]

Supplemental Materials

1. Reagents, Cell Culture, Antibodies, and Plasmid Preparation

Heteroantibodies against β-Actin (AC-15), $p16^{Ink4a}$ (N-20), and GFP (06-896) were obtained from Santa Cruz biotechnology Inc. (Santa Cruz, CA, USA) and Millipore Inc. (Billerica, MA, USA), respectively. Monoclonal antibody against JDP2 (#176) was prepared as described previously [1]. Sulforaphane (SNF) was purchased from the LKT laboratory (St. Paul, MN, USA). Human DAOY, HeLa $CD4^+$ and 293T cells were obtained from ATCC (Manassas, VA, USA), and cultured in Dulbecco's Modified Eagle Minimal Essential medium (DMEM)-Ham's F-12 (Invitrogen, Grand Island. NY, USA). Wild-type (WT) and *Jdp2* KO MEFs were prepared as described elsewhere [1,2]. The full-length plasmids pcDNA3-rat Nrf2 and pcDNA3–rat MafK were kindly provided by Dr. T Nguyen (Schering-Plough Research Institute, Kenilworth, NJ, USA). The FLAG-Nrf2, FLAG-MafK and FLAG-JDP2 were generated by amplification by PCR and cloned into pCMV_S-FLAG vector (RIKEN DNA Bank, Tsukuba, Japan). All recombinants were confirmed by DNA sequencing.

2. Transient Ttransfection and Luciferase Reporter Assay

WT and *Jdp2* KO MEFs (1×10^5 cells) were plated into each well of 12-well plates and cultured for 24 h. The cells were then cotransfected with indicated amount of pGL4-hQR25-firefly luciferase reporter and pGL4-TK plasmid encoding *Renilla* luciferase (Promega, Madison, WI, USA) using the Effectene® transfection reagent kit (Qiagen, Valencia, CA, USA). After 24 h of incubation, the cells were incubated in the presence or absence of SFN in DMSO (or DMSO alone, as a control) for 48 h. The activities of luciferase and *Renilla* luciferase were measured in a luminometer (Berthold Technologies Gmbh and Co. KG, Bad Wildbad, Germany) using the Dual-Luciferase Reporter Assay System (Promega). Luciferase activity values were normalized to transfection efficiency as described elsewhere [2].

3. Generation of Jdp2 Promoter-Cre Driver Mice and Genotyping

The minigene contained a 2.5 kb DNA fragment of 5'-end of the promoter region of *Jdp2*, the transcriptional initiation site preceding the ATG starting codon of *Jdp2* mRNA, a DNA fragment of nuclear localization signal (NLS) of the *Cre* gene, a DNA fragment of internal ribosome entry site (IRES), and the NLS-LacZ (β-galactosidase) gene followed by an SV40 polyadenylation signal (polyA). The minigene was released from the plasmid by digestion with *Swa*1 and was used for microinjection of fertilized FVB/N eggs. Twenty-five independent transgenic mouse lines (F_o) were obtained and crossed with ROSA 26R reporter [3] or ZEG (TCTB-βgeo–Green Fluorescein Protein) reporter [4] mice. Functional F_1 offspring from each transgenic line were identified by X-gal staining or GFP fluorescence. The genotyping of these F1 founders was examined. Mice were maintained on C57BL6 × 129v, 129v congenic, and C57BL/6J congenic backgrounds and are available from the RIKEN BioResource Center (RIKEN BRC) at Tsukuba, Japan, and National Laboratories of Animal Center (NLAC) in Taipei, Taiwan. All animal procedures were approved by the RIKEN BRC center and NLAC. PCR genotyping was performed with the following three primers: Primer1, 5'-GGGTTAAGTGGAATCAGTTCTGCTC-3'; Primer 2, 5'-GGTTCAGGGGGAGGTGTGGGAGG-3' (SV40 polyA); Primer 3, 5'-GGAAGGCGATCCCATAGGAAGAG-3'. The sizes of the WT (Primers 1 and 3) and mutant fragments (Primer 1 and Primer 3) were 688 and 420 bp, respectively. Embryos

and organs were dissected into ice-cold PBS and visualized for GFP fluorescence as described below. For tissue sections, samples were embedded directly into OCT (Tissue-Tek, Sakura, Zoeterwoude, The Netherlands), frozen at –70 °C and cryosectioned at 7 μm onto poly-L-lysine-coated slides. After drying for 1–4 h at room temperature, the slides with sections were stored at –20 °C. For EGFP, the slides were mounted directly with Cytoseal mounting solution (Thermo Fischer Scientific, Waltham, MA, USA). For LacZ staining, slides were fixed for 5 min in 0.2% glutaraldehyde and washed three times for 5 min in LacZ wash buffer (2 mM $MgCl_2$, 0.01% sodium deoxycholate, 0.2% Nonidet P40 in 100 mM PBS). The slides were then stained in LacZ staining solution (0.5 mg/mL X-gal, 5 mM potassium ferrocyanide, and 5 mM potassium ferricyanide in LacZ wash buffer) for 4–6 h at 37 °C while protected from light. When the staining was complete, the slides were rinsed in PBS, dehydrated through a graded ethanol series, and mounted with coverslips. For embryos and whole-mount organs, green fluorescence was observed on a Leica Wild M3C stereo microscope with an MAA-02 Universal light source from BLS-Ltd. (www.bls-ltd.com, Budapest, Hungary). These sections were viewed on a Leitz DMRB (San Jose, CA, USA) fluomicroscope equipped with epifluorescence lighting, using the FITC filter set. All microscope samples were photographed using a CoolSnap digital camera.

4. Western Blot Analysis

Cells were harvested using a modified RIPA buffer (10 mM Tris-HCl, pH 8.0, 150 mM NaCl, 1 mM EDTA, 0.1% NP-40, 1% deoxycholate, 50 mM sodium fluoride, 50 mM sodium orthovanadate, and 1 mM PMSF) and a protease inhibitor cocktail (Nacalai Tesque, Kyoto, Japan). The preparation of cell lysates, SDS-PAGE (10% or 12% gel) and western blotting were performed as described elsewhere [1].

5. Generation of DAOY iPSCs

Human *OCT3/4*, *SOX2*, *KLF4*, and *c-MYC* (human "four-factors") cDNAs were amplified by reverse transcription-PCR (RT-PCR) using mRNA prepared from hES cells as the template with the primer sets as described previously [5]. The cDNAs were inserted into the pENTR/D-TOPO entry vector plasmid (Invitrogen) and verified by sequencing. The cDNAs in pENTR/D-TOPO were then transferred to the pCSII-EF-MCS-IRES2-Venus lentiviral vector plasmid using Gateway LR clonase (Invitrogen). Lentiviral vectors pseudotyped with the vesicular stomatitis virus G glycoprotein (VSV-G) were produced by transient transfection of three plasmids into 293T cells (ATCC): the packaging plasmid (pCAG-HIVgp), the vesicular stomatitis virus G glycoprotein- and Rev-expressing plasmid (pCMV-VSV-G-RSV-Rev), and the lentiviral vector plasmid. The culture supernatant was concentrated by ultracentrifugation, and the viral pellet was resuspended in HBSS. The titers of vectors were determined by infection of HeLa $CD4^+$ cells with serial dilutions of the vector stocks followed by fluorescence-activated cell sorting analysis for $Venus^+$ cells. DAOY cells were cultured overnight in DMEM (Invitrogen) supplemented with 10% FBS-containing lentiviruses at a multiplicity of infection of 30 in flat-bottomed 24-well plates at 37 °C under 5% CO_2 in air. Two days after transduction, the cells were harvested by trypsinization; 5×10^5 cells were replated into 100-mm culture dishes and cultured on mitomycin C-treated mouse embryonic fibroblasts at a concentration of 5×10^6 at 37 °C under 5% CO_2 and 5% O_2 in air. Hypoxic conditions were sustained for 14 days. The culture medium of iPSC (iPSM) comprised 78% DMEM-Ham's F-12 supplemented with 20% knock-out serum

replacement (KSR, Invitrogen), 2 mM GlutaMax (Invitrogen), 1% nonessential amino acids, 0.1 mM β-mercaptoethanol, 10^3 units/mL human LIF (leukemia inhibitory factor; Invitrogen), and 4 ng/mL human recombinant basic fibroblast growth factor (bFGF) (Wako, Osaka, Japan). On days 12–18, rabbit ESC-like cell colonies were isolated mechanically and replated onto mouse embryonic fibroblasts. iPSCs were passaged by incubating the cells with detaching solution [0.25% trypsin (w/v), 0.1 mg/mL collagenase IV, 20% KSR, 1 mM $CaCl_2$] for 1 min at room temperature and mechanically disaggregating the resulting small clumps into single cells. Cells were then counted in a hemocytometer, resuspended, and plated in iPSM supplemented with 8 ng/mL bFGF. Fresh medium was added daily and cells were passaged every 3 days. Colonies were selected for staining for alkaline phosphatase and stem cell markers, and analyzed further. Alkaline phosphatase staining was performed with the ES cell Characterization Kit (Chemicon Millipore) according to the manufacturer's protocol.

6. sh-RNA and Adenovirus Vectors

Vectors encoding shRNA targeting human $p16^{Ink4a}$ has been described elsewhere [6]. Adenovirus-Jdp2 vector was constructed by inserting JDP2 cDNA into pAxCAwt [7], and infectious viral particles were produced and purified as described elsewhere [7]. DAOY cells in 6 well plates (1×10^6 cells/well) were infected with lentivirus OCT4 at m.o.i. of 2 and YFP signal was detected to confirm the full infection. OCT4-DAOY cells on mitomycin C treated SNF feeder cells again infected with Ad-GFP, Ad-Jdp2 at m.o.i of 3 in the presence of DMEM plus 0.05% FCS, or lentivirus shRNA-$p16^{Ink4a}$ or control vector only. Two days after infection, the medium was replaced with DMEM plus 10% FCS, and the cells were harvested after further incubation for 2 weeks to generate the iPSCs, and stained each iPSCs colonies as described in the Experimental Section.

Figure S1. Time course of ARE-luciferase activity after exposure with SFN. WT and *Jdp2* KO MEFs (5×10^4) were transfected with 400 ng of pGL4–hQR25–luciferase as described in Experimental section. One day after transfection, cells were exposed to SFN (5×10^{-6} M) and incubated until 48 h. Relative luciferase activity was measured at indicated incubation time. Luciferase activity of *Jdp2* KO MEF was taken as 1.0. Each value represents the mean ± SD (n = 3). $p < 0.05$.

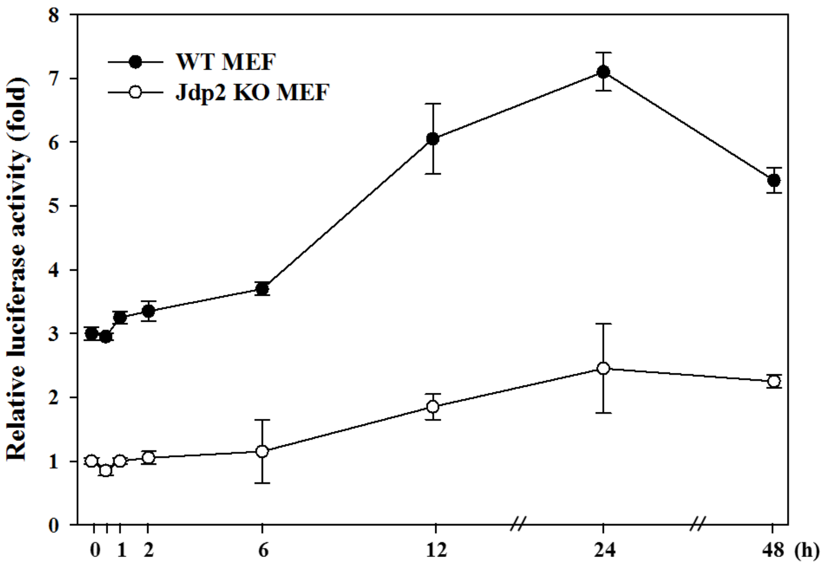

Figure S2. Generation of *Jdp2* promoter-Cre mice and the GFP expression is restricted in cerebellar granule cells. (**A**) Scheme of the expression vector used for generation of the transgenic line of *Jdp2*-Cre. The *Jdp2*-promoter Cre vector consists of 2.5 kb *Jdp2* upstream promoter region of Mouse *Jdp2* gene, the NLS-Cre, IRES, NLS-Lac Z and a 3' SV40 intron/polyadenylation signal sequence (poly A). (**B**) Adult organs are displayed (from center to the edge): brain, liver, testis or ovary (top rows); lung, muscle, intestine (2nd rows); stomach, thymus, spleen (3rd rows); kidney, leg with bone, intestine (bottom rows). GFP was detected in the brain, especially the cerebellum. GFP signal in the cerebellum region was detected by antibody against GFP in *Jdp2*-Cre × Z/EG (p24) double transgenic mice (bottom panel). The dorsal and ventral views are shown respectively. The cerebellar granule cells were detected (**a**). (**b**) and (**c**) showed the Lac Z stained cells and we do not know the exact cell-types. Scale bars; 100 µm.

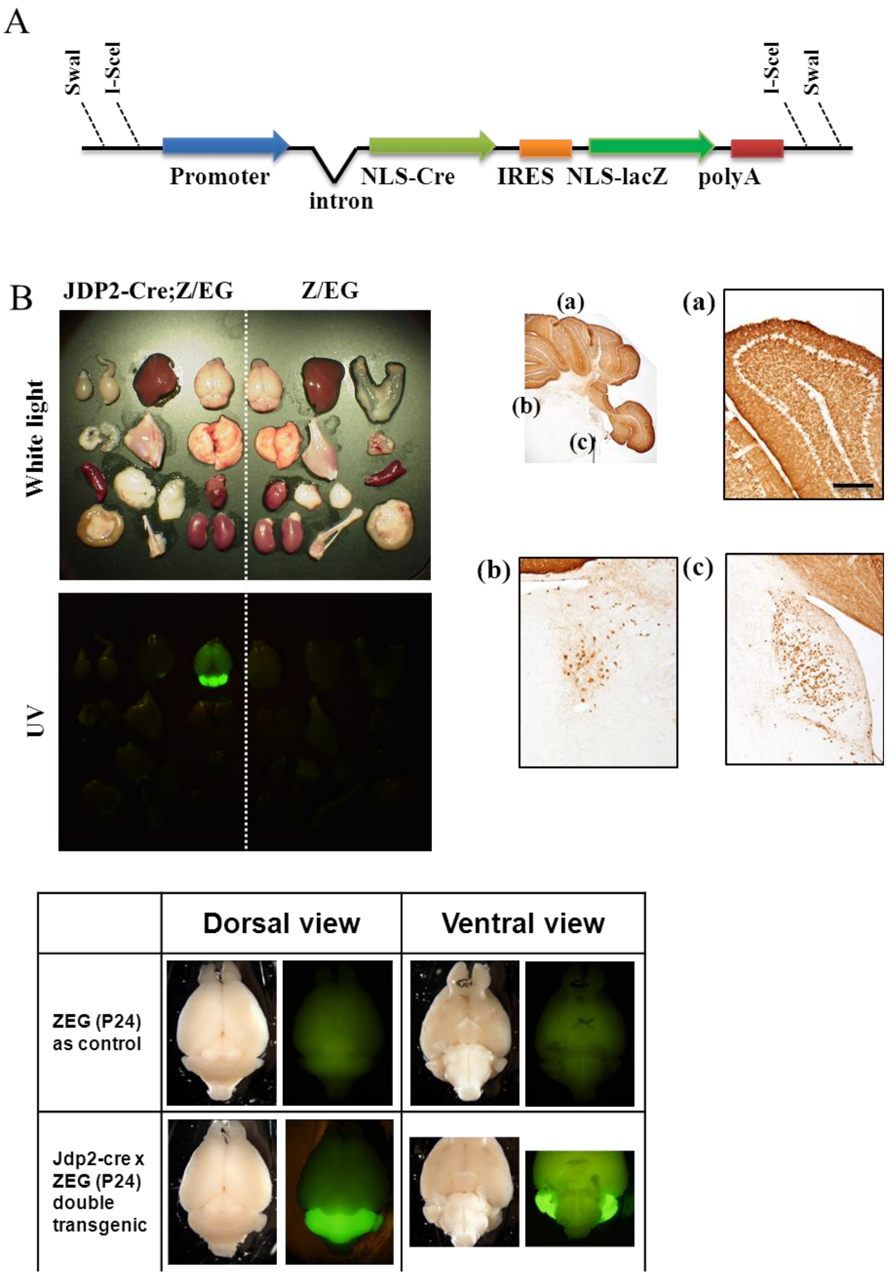

Figure S3. Protein detection in DAOY, SNL feeder cells, and 1F-DAOY transformed clones, 1F-DAOY transformed clones without SNL feeder cells using Western blotting. The cellular lysates (25 µg) were separated on 12% acrylamide-SDS-PAGE and then transferred onto a membrane. JDP2 (#176), β-ACTIN (AC-15) were immunodetected using respective antibodies. Lane 1; SNL feeder cells, lane 2, DAOY parent cells, lane 3; 1F-DAOY clones which were infected with lentivirus encoded JDP2 with SNL feeder cells (1FV), and lane 4; 1F-DAOY clones which were infected with lentivirus encoded JDP2 without SNL feeder cells (1FVN).

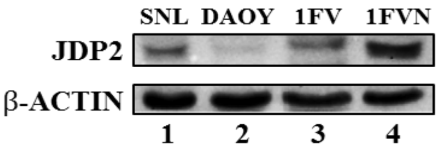

Figure S4. Comparison of normoxia and hypoxia to generate the alkaline phosphatase-positive iPSCs-like cells. OCT4-DAOY cells was infected by adenovirus encoded JDP2 or GFP, and transduced by lentivirus sh-RNA-p16^{Ink4a} or lentivirus sh-RNA vector only. These cells were grown in culture conditions compatible with pluripotent stem cell growth under the normaxia (20% O_2) and hypoxia (3% O_2). Colonies was counted by the alkaline phosphatase staining as described in Experimental section. (**A**) The number of positive colonies is shown as the mean ± SD of three representative experiments. Each value represents the mean ± SD (n = 3). $p < 0.05$. (**B**) Western blot analysis of JDP2, GFP, p16^{Ink4a} and β-Actin in various treated DAOY iPSC-like cells, and lentivirus sh-RNA-p16^{Ink4a} and control lentivirus sh-RNA vector infected DAOY iPSC-like cells. JDP2 (#176), GFP (06-896), p16^{Ink4a} (N-20), and β-ACTIN (AC-15) were immunodetected using respective antibodies. β-Actin is the internal control. Left panel, lane 1; OCT4-infected original DAOY cells, lane 2; Ad-JDP2 or Ad-GFP or mock infected OCT4-DAOY iPSC-like cells. Right panel, lane 1; OCT4-infected original DAOY cells, lane 2; lentivirus sh-RNA-p16^{Ink4a} infected iPSC-like DAOY cells, lane 3; lentivirus control sh-RNA-vector infected iPSC-like DAOY cells.

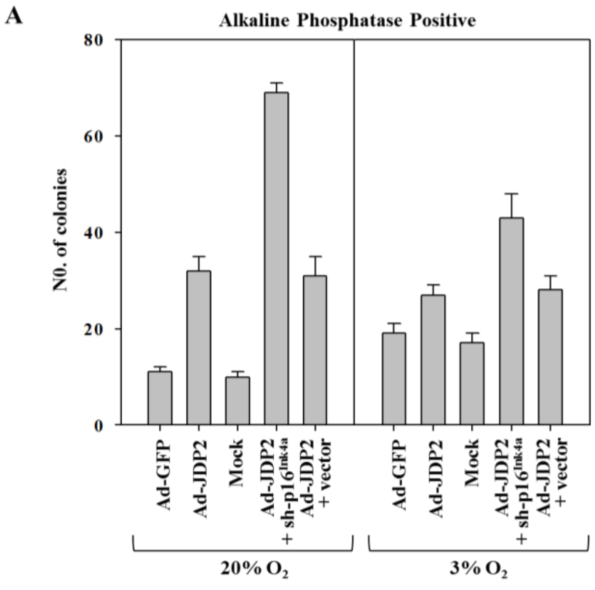

Figure S4. *Cont.*

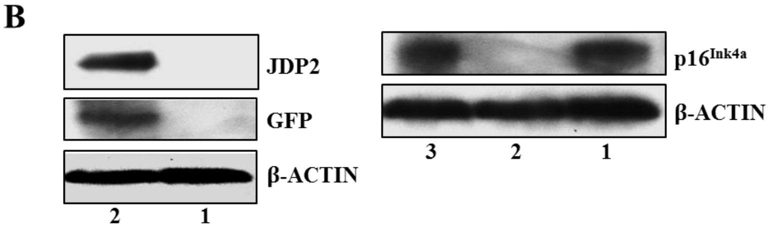

References

1. Pan, J.; Nakade, K.; Huang, Y.C.; Zhu, Z.W.; Masuzaki, S.; Hasegawa, H.; Murata, T.; Yoshiki, A.; Yamaguchi, N.; Lee, C.H.; *et al*. Suppression of cell-cycle progression by Jun dimerization proten-2 (JDP2) involves downregulation of cycling-A2. *Oncogene* **2010**, *29*, 6245–6256.
2. Hou, D.X.; Fukuda, M.; Johnson, J.A.; Miyamori, K.; Ushikai, M.; Fujii, M. Fisetin induces transcription of NADPH:quinone oxidoreductase gene through an antioxidant responsive element-involved activation. *Int. J. Oncol.* **2001**, *18*, 1175–1179.
3. Soriano, P. Generalized lacZ expression with the ROSA26 Cre reporter strain. *Nat. Genet*. **2006**, *21*, 70–71.
4. Novak, A.; Guo, C.; Tang, W.; Nagy, A.; Lobe, C.G. Z/EG, a double reporter mouse line that expresses enhanced green fluorescent protein upon Cre-mediated excision. *Genesis* **2000**, *28*, 147–155.
5. Ohnuki M.; Takahashi, K.; Yamanaka, S. Generation and characterization of human induced pluripotent stem cells. *Curr. Protoc. Stem Cell Biol.* **2009**, doi:10.1002/9780470151808.sc04a02s9.
6. Barradas, M.; Anderson, E.; Acosta, J.C.; Li, S.; Banito, A.; Rodriguez-Niedenfuhr, M.; Maertens, G.; Banck, M.; Zhou, M. M.; Wash M. J.: *et al. Genes Dev.* **2009**, *23*, 1177–1182.
7. Kanegae, Y.; Lee, G.; Sato, Y.; Tanaka, M.; Nakai, M.; Sugano, S.; Saito, I. Efficient gene activation in mammalian cells by using recombinant adenovirus expressing site-specific Cre recombinase. *Nucleic Acids Res.* **2006**, *23*, 3816–3821.
